# PTIGS-IdIt, a system for species identification by DNA sequences of the psbA-trnH intergenic spacer region

**DOI:** 10.1186/1471-2105-12-S13-S4

**Published:** 2011-11-30

**Authors:** Chang Liu, Dong Liang, Ting Gao, Xiaohui Pang, Jingyuan Song, Hui Yao, Jianping Han, Zhihua Liu, Xiaojun Guan, Kun Jiang, Huan Li, Shilin Chen

**Affiliations:** 1 The Key Laboratory of Bioactive Substances and Resources Utilization of Chinese Herbal Medicine, Ministry of Education, Institute of Medicinal Plant Development, Chinese Academy of Medical Sciences & Peking Union Medical College, Beijing 100193, P. R. China; 2 School of Computer Science & Engineering, Beihang University, 37 Xueyuan Road, Haidian District, Beijing 100191, P.R.China; 3 College of Life Sciences, Qingdao Agricultural University, Qingdao 266109, P.R. China; 4 Center for BioInformatics, University of North Carolina at Chapel Hill, Chapel Hill, NC 27599, USA; 5 Pidit Ltd, 192 West Grant Ave, Edison, NJ 08820, USA

## Abstract

**Background:**

DNA barcoding technology, which uses a short piece of DNA sequence to identify species, has wide ranges of applications. Until today, a universal DNA barcode marker for plants remains elusive. The *rbc*L and *mat*K regions have been proposed as the “core barcode” for plants and the ITS2 and *psbA-trnH* intergenic spacer (PTIGS) regions were later added as supplemental barcodes. The use of PTIGS region as a supplemental barcode has been limited by the lack of computational tools that can handle significant insertions and deletions in the PTIGS sequences. Here, we compared the most commonly used alignment-based and alignment-free methods and developed a web server to allow the biologists to carry out PTIGS-based DNA barcoding analyses.

**Results:**

First, we compared several alignment-based methods such as BLAST and those calculating P distance and Edit distance, alignment-free methods Di-Nucleotide Frequency Profile (DNFP) and their combinations. We found that the DNFP and Edit-distance methods increased the identification success rate to ~80%, 20% higher than the most commonly used BLAST method. Second, the combined methods showed overall better success rate and performance. Last, we have developed a web server that allows (1) retrieving various sub-regions and the consensus sequences of PTIGS, (2) annotating novel PTIGS sequences, (3) determining species identity by PTIGS sequences using eight methods, and (4) examining identification efficiency and performance of the eight methods for various taxonomy groups.

**Conclusions:**

The Edit distance and the DNFP methods have the highest discrimination powers. Hybrid methods can be used to achieve significant improvement in performance. These methods can be extended to applications using the core barcodes and the other supplemental DNA barcode ITS2. To our knowledge, the web server developed here is the only one that allows species determination based on PTIGS sequences. The web server can be accessed at http://psba-trnh-plantidit.dnsalias.org.

## Background

DNA barcoding technology uses a short piece of DNA sequence to identify species. In animals, a region of the *COI* gene has been found to possess properties like high PCR amplification efficiency, high DNA sequencing success rate, and high discrimination power. Consequently, the gene has been selected as the universal DNA barcode. However, in plants, no DNA sequences have been found to be comparable to the *COI* in animals. Several DNA regions such as *rbcL*, *matK*, ITS2, ITS, and *psbA-trnH* have been proposed as potential candidates for plant DNA barcodes [1-6]. The *psbA-trnH* intergenic spacer (PTIGS) region has been successfully used to resolve problems in species identification in a number of studies [3,7-10]. As one of the supplemental barcodes, PTIGS has several favorable characteristics. First, it can be easily amplified across a broad range of land plants. Based on the sequences of the conserved coding regions of *psbA* and *trnH*, universal primers can be easily designed to amplify the PTIGS region. Secondly, the insertions/deletions of the PTIGS region are common and lead to various length among different plant groups. As a result, PTIGS has the highest percentage of nucleotide difference and micro-inversions and it has become the most variable plastid region in some group of plants [3,9,11].

Various methods have been tested on simulated or specific datasets. Using available gymnosperm nrITS2 and plastid *mat*K sequences as a test dataset, Little et al. have estimated the precision and accuracy of hierarchical clustering methods (parsimony and neighbor joining), similarity methods (BLAST, BLAT, and megaBLAST), combined clustering/similarity methods (BLAST/parsimony and BLAST/neighbor joining), diagnostic methods (DNA–BAR and DOME ID) for species identification [12]. It was found that no method was able to accurately identify query sequences for species at a frequency that would be considered useful for routine specimen identification. The success rate for unambiguously correct identification ranges from 42% to 71%. More recently, phylogenetic and statistical classification methods such as neighbor-joining (NJ), PhyML, k-Nearest Neighbor (k-NN), Classification and Regression trees (CART), Random Forest, and Kernel have been compared based on their performance on DNA barcode analyses using simulated and specific datasets [13]. The authors found that no method is superior to others, although the simplest method of all, “one nearest neighbor” was found to be the most reliable. Because of wide-spread phenomena of monophyly and paraphyly in plant kingdom, there are no defined borders for many plant species. Consequently, classification based methods such as Support Vector Machine (SVM) have not been considered more effective than the nearest neighbor method. In addition, several studies have applied the alignment free methods on DNA barcode analyses using *COI*[14] or ITS2 [12] as the barcode markers, even though it is arguable whether these studies are necessary as *COI* has little indels and less difficulty in sequence alignment. It has not been determined whether or not they can be extended to other markers.

PTIGS sequence has unique characteristics that make it an important supplemental plant DNA barcode. However, its usage has been limited by the lack of computational tools that allow the easy retrieval and annotation of the PTIGS sequences, and the subsequent species identification due to the difficulty in aligning PTIGS sequences. In this study, we have developed a web server system that tackles these problems, which, hopefully, will facilitate PTIGS-based DNA barcoding analyses.

## Implementation

The methods and their combinations were implemented using C and Perl programming languages. Perl Catalyst framework was then used to develop the PITGS-IdIt web server. All source codes are available upon request. The performance of the various methods was measured on a machine with 24G RAM, 8 processors, running Red Hat 4.1.2-46 operating system.

## Results

### Algorithms

The current web server has three main functions: (1) retrieval of PTIGS sequences, (2) annotation of novel PTIGS sequences, and (3) species identification based on PTIGS sequences. To allow the easy retrieval of PTIGS sequences, we downloaded all sequences from the PTIGS loci in GenBank (Release 178), available from Additional File 1. Each sequence was divided into the 5’ *psbA* region, the core PTIGS region, and the 3’ *trnH* region based on GenBank annotations. Different sequences were grouped at various taxonomy levels of class, order and family, genus, and species, available from Additional File 2. For each species, the sequences of all the PTIGS core regions were assembled using Phrap program (version 0.990319) to generate the consensus sequence, which is considered to be the reference sequence for each species. All processed sequences are stored in two dimensions, the sequence regions (the 5’, core, and 3’) and the taxonomy levels (class, order, family, genus, and species). The users can easily retrieve any sequence in the two dimensions. For more details, please see the corresponding application page.

We found manual annotation of PTIGS regions, that is, defining the 5’, core, and 3’ regions can be extremely tedious. To automate this process, we implemented a Hidden Markov Model (HMM)-based annotation engine. To use this engine, the users need to prepare two multiple alignments of sequences for the 5’ and 3’ regions respectively. HMM models are built using hmmbuild (HMMER 2.0) [15,16]. The models are then used to scan the sequences to be annotated using hmmsearch (HMMER 2.0). Last, a parser written in Perl identifies the regions in the sequences that were found to match the 5’ and 3’ HMM models, completing the annotation process.

As described above, one particular characteristic of the PTIGS regions is the presence of significant portions of indels [3,7,17-19], causing difficulties in multiple sequence alignment. These indels actually contain the information that can be used to distinguish the two sequences, which has been ignored by alignment-based methods such as BLAST and P distance method (P) [20-22]. Consequently, we are set to find methods that include the indels in the comparison of two PTIGS sequences. To evaluate the performance of these methods, we first constructed a dataset from species that has at least two PTIGS sequence. This produced a dataset containing 11137 PTIGS core region sequences (Table 1).

**Table 1:** Taxonomy coverage of PTIGS sequences.

Category	Families	Genera	Species	Samples	Related taxids
Angiosperms	149	644	1961	9404	3398
Dicotyledons	108	451	1367	7468	71240
Monocotyledons	30	146	472	1603	4447
Ferns	12	19	42	124	3290
Gymnosperm	8	16	65	206	3312, 58020, 58021, 58022
Mosses	34	46	72	287	3208

The number in each cell represents the number of taxonomy units at the corresponding taxonomy levels for the particular group used in our analyses. Essentially they correspond to the taxonomy units whose species having at least two sequences. The sequences can be downloaded from the web server.

After reviewing various methods, the one that calculates the Edit (E) distance [23,24] was selected. This method uses dynamic programming techniques to align two sequences [25], then calculate the number of steps (i.e. Edit distance) needed to transform one sequence into the other. The allowed transformation steps include transition (i.e. converting one nucleotide to another), insertion and deletion. An alternative solution to include unaligned regions in sequence comparison is to use the alignment-free methods as described before [12,14]. In this study, we implemented such a method by first converting input sequences into vectors of frequencies of kmers (i.e. short oligonucleotides of size K). Then the pearson correlation coefficient was calculated for each pair of vectors as their distance. To find the best method for PTIGS-based species determination, the E and kmer-based methods were compared with the other commonly used methods for species discrimination, namely, BLAST and P distance method. Because the calculation of the P or Edit distances between the query and all sequences in the reference databases is prohibitively time-consuming, we combined these base methods and compared their success rate and performance.

We first tested the success rate for discriminating PTIGS sequences under various kmer sizes to determine the optimal size for the kmer based method. As shown in Table 2, vectors of frequencies of di-nucleotide were found to have sufficient discrimination power (success rate being 0.761) with a significant less processing time (95.3 mS) comparing to those of other kmer sizes. Consequently, we use kmer size of 2 in the following study and named the method Di-nucleotide Frequency Profile (DNFP).

**Table 2:** Discrimination success rate and performance using the kmer-based method at different Kmer sizes

Size of Kmer	Discrimination success rate	Performance (cpu time cost : mS)
2	0.761	95.3
3	0.775	390.4
4	0.795	1564.2
5	0.823	5060.4
6	0.813	19007.1
7	0.611	74949.3

The test data is the same as those shown in Table 1.

The taxon coverage of the test dataset is shown in Table 1. Each species in the dataset contains at least two individual sequences. Each of the sequences was used as the query to search against the database with (labeled as “include self” in Table 3) or without the query sequence itself (labeled as “not include self” in Table 3) using the corresponding methods. Previous studies have showed that the prerequisite for meaningful DNA barcoding analysis is that the reference database must contain the query sequences or sequences from the same species as the query sequence. When using the database that is “include self”, the query sequence will always pick up itself from the reference database. The question is whether or not it picks up non-self sequences at the same time. If it only picks up its own sequence, it is considered a success. If it picks up non-self sequence at the same time, it is considered a failure. For the database that is “not include self”, it contains other sequences from the same species but not the query sequence itself. When using this database, if the nearest neighbors only contain sequences from the same species, the identification is considered a success, otherwise it is considered a failure.

**Table 3:** Discrimination success rates and performance using various method combinations for the dataset containing all sequences shown in Table 1.

	Include				Not include			
	
Method	Correct	Wrong	Ratio	Time	Correct	Wrong	Ratio	Time
B	6291	4846	0.5649	0.4213	5323	5814	0.4780	0.5653
B+P	7744	3393	0.6953	5.0552	6496	4641	0.5833	6.4200
B+E	8650	2487	0.7767	36.7524	7034	4103	0.6316	52.3093
D	8477	2660	0.7612	0.2496	6669	4468	0.5988	0.5347
D+P	8477	2660	0.7612	2.3828	6670	4467	0.5989	2.4413
D+E	8687	2450	0.7800	21.5453	7363	3774	0.6611	15.6762
B+P+E	8651	2486	0.7768	12.9270	7096	4041	0.6372	11.6186
D+P+E	8686	2451	0.7799	9.8835	7401	3736	0.6645	9.7989

Ratio indicates the number of correctly identified/total number of tests. The performance shows the average time in second taken to complete a query. The base methods are B: BLAST; P: P Distance; E: Edit Distance; D: DNFP. “Included” means that the query sequences are included in the reference database, while “excluded” means that the query sequences are not included in the database when performing the analyses.

To identify the best methods for discriminating PTIGS sequences, we construct eight different methods based on the four base methods BLAST (B), P distance method (P), Edit distance method (E) and DNFP method (D), which are B, B+P, B+E, D, D+P, D+E, B+P+E and D+P+E respectively. Each query sequence was compared to each sequence (target) in the reference database to obtain the corresponding BLAST distance (represented by similarity e-value or score), P distance, Edit distance or DNFP distance (represented by the pearson correlation between the kmer frequency vectors of the query sequence and target sequence. The target sequence(s) that has the smallest distance (i.e. “nearest neighbor”) was considered the hit. “B+P” means that if the query sequences cannot be successfully identified using B method, it will be subjected to P method for further identification. The meanings for other methods are similar.

The success rate and performance for each method combination against two different databases are shown in Table 3. We found that the discrimination success rate follows E > DNFP > P > B. In terms of speed, it follows that DNFP > B > P > E, from the fastest to the slowest. The DNFP method is the fastest and has an identification success rate close to the highest rate (~80%), which is achieved when the E method was used. The best method exceeds the poorest method (B) by a margin of ~20% in terms of success rate. The slowest method on the other hand, which involves E method, is about 20 times slower than the fastest method (DNFP).

To demonstrate the usefulness of this web server and its identification methods, we use a query sequence with accession number EF590731 (*Polytrichum juniperinum*; taxid 129213) as an example. Using Blast search method, three sequences (sFigure 1 and 2) were found to have the smallest e-value, they are GQ248374 (taxid: 129213), EF590730 (*Polytrichum commune*; taxid: 3213) and EF590731 (taxid: 129213). The Blast results are shown in Table 4. The query sequence was then predicted to belong to species *P. juniperinum* (taxid: 129213) or *P. commune* (taxid: 3213). This result is considered unsuccessful. However, using Blast+P distance method, the distances between the query sequence and the three sequences (available from Additional File 3) with the best e-value scores are shown in Table 5. As shown, the query sequence was successfully identified to belong to *P. juniperinum.* The query sequence, the three best match sequences, the multiple alignments are shown in supplementary Figure 1 and 2 respectively. A quick tutorial is also available on the “identify” web site using this sequence as an example.

**Table 4:** Species identification using Blast method for an exemplar query sequence.

Query	Target	% similarity	QS*	QE*	TS*	TE*	E value	Score
Query	GQ248374_129213	100.00	1	61	20	80	4e-32	121
Query	EF590730_3213	100.00	1	61	1	61	4e-32	121
Query	EF590731_129213	100.00	1	61	1	61	4e-32	121

*QS: Query Start; *QE: Query End; *TS: Target Start; *TE: Target End.

**Table 5:** Species identification using Blast+P method for an exemplar query sequence.

Query	Target	K2P distance
query	EF590731	0.000000
query	EF590730	0.027541
query	GQ248374	0.000000

### Application pages

#### Review the reference barcode sequence

This page provides a summary for the PTIGS region of each species. Usually, there are many individual sequences associated with a single species, and the consensus sequence is an ideal way to represent the “average” DNA barcode sequence of a species. The input is the taxid from NCBI for a species. While there are many taxonomy systems available, we use NCBI’s taxonomy system in our web server because the primary species identifier in NCBI’s system (i.e. taxid) is directly linked to DNA sequences. We decided not to duplicate this taxonomy system and the user will have to go to NCBI’s taxonomy page (http://www.ncbi.nlm.nih.gov/taxonomy) to find the taxid for the taxonomy groups of interest. After the query has been submitted, this module will return all sequences associated with this taxid as well as the reference sequence for the species under query. The taxid and sequence accession numbers are linked to their records in GenBank which allows the users to obtain more detailed information about the species and the sequences.

#### Retrieve different segments of the PTIGS locus

We defined the PTIGS locus as composed of the 5’ *psbA* region, the PTIGS core region, and the 3’ *trnH* region. The sequences of the PTIGS core region should be the region used for DNA barcoding. This page allows the user to retrieve a particular region as well as the entire locus region that include the 5’, core and 3’ regions. While the core region is needed for species determination analysis, the 5’ and 3’ regions can be used to annotate newly obtained PTIGS sequences (see below). The input is similar to the view page, which contains a single taxid. After specifying the regions of interest, this module will return all sequences associated with the query taxid for the corresponding region.

#### Annotate novel sequences based on Hidden Markov Model (HMM)

As described above, the PTIGS locus consists of three regions: the 5’ *psbA*, the PTIGS core, and the 3’ *trnH* regions. This module allows the determination of these three regions for unannotated PTIGS locus sequences using HMM. Three input files are needed for this module. The first file should contain multiple alignments of sequences from the *psbA* region. The second file should contain multiple alignments of sequences of the *trnH* region. The third file contains the sequences to be annotated. The user will have to provide the 5’ and 3' sequences that are needed to build the model. It is recommended that the length of the aligned sequence fragments should be between 20-30 bp and the total number of sequence fragments should be >10, in order to build the HMM. The backend annotation engine will return the sequences with the three regions determined.

#### Species identification based on a PTIGS sequence

This page provides the interface for our species identification engine which consists of the eight methods described above. Only one sequence is accepted at this time for species determination. After selecting the method of interest, the sequence will be compared to the backend reference database using the specified method. We use “nearest neighbor” as the criterion for species inference because this has been found to be the most reliable. The nearest neighbor is the sequence(s) in the database that has or have the smallest BLAST distance, P distance, Edit distance or DNFP distance to the query. Consequently, the nearest neighbor to the query sequence from the backend will be identified and considered as the most likely identity of the input sequence. The taxid or taxids, if there are multiple hits, will be returned along with the time taken to complete the query.

#### Check the performance for 8 different identification methods for given operational taxonomy units

As described in the algorithm section, eight different methods have been developed. These methods were first applied to a comprehensive and heterogeneous dataset (Table 1). Subsequently, they were applied to datasets containing sequences from various taxonomy groups including kingdom, phylum, class, order, and family. The rationale behind this is that it would be very valuable for the biologists who work on a particular group of plants to know if the PTIGS core region is a good DNA barcoding marker for the taxonomy group they are studying. One can simply select the group of interest from the pull down list and review the success ratio and performance of the eight different methods for the group of interest. However, we realize that particular taxonomy group of interest might not be found in the list. This is usually because no sequences are available for this group when the data were compiled. It should be noted that the analyses were carried out only on species that has at least two sequences because for species having only one sequence, the “not include self” analysis cannot be performed, and consequently, the comparison between the “include self” and “not include self” analyses is not applicable.

## Discussion

So far, two core barcodes and two supplemental barcodes have been proposed for DNA barcoding of plant species. To optimize the usage of these barcodes, each barcode and the relevant methods have to have been first studied in-depth. The lack of computational tools that facilitate the retrieval and annotation of PTIGS sequences, and that maximize PTIGS-based species determination has hindered its use as a supplemental DNA barcode. In this study, we have developed a web server system that tackled these problems. Particularly, we have constructed several hybrid methods that would increase the species discrimination rate and also have acceptable performance.

We construct the eight methods for the following reasons. Calculating BLAST distance requires local alignment. Its calculation only uses the nucleotides within the locally aligned regions. In contrast, calculating both P and Edit distances require global alignment. P distance is calculated using only nucleotides within the globally aligned regions. On the other hand, the calculation of Edit distance uses all nucleotides between the two sequences. Consequently, among the alignment-based method, the Edit distance method utilizes all available sequence variation information and should have the highest discrimination power. However, the cost for increased resolution is the rapid degradation of performance. We combined the base methods to form B+P, B+E and B+P+E so that fast methods can be applied first and the queries that can not be successfully identified can then be subjected to more sensitive but slower methods. The alignment-free method DNFP is most accurate and fastest (Table 3). However, totally unrelated sequences might have similar DNFP. We combined D+P, D+E and D+P+E to further resolve the queries that cannot be resolved by DNFP method alone.

We include all eight methods in the web server for the following considerations. In a typical scenario, the user can employ the DNFP method first for quick species identification. He can further drill down with more sensitive methods if the query fails to be assigned to a single species. In addition, the alignment-based methods can be used to validate the results obtained from the DNFP method.

The methods developed here have significantly increased the success rate of PTIGS-based species identification when using the BLAST method. These methods can be easily extended to other DNA barcodes. However, it should be emphasized that the improvement of methods will not be able to solve many problems inherent to DNA barcoding technologies. For example, computational methods can differentiate species for a given marker only if there are variations in the marker sequences among different species. In addition, species determination is only useful when the query sequences exist in the reference database. When the query sequence is not in the reference database, the identification can be rather unreliable. Last, these methods would be useless in the cases that the voucher samples are wrongly-identified, or the sequence annotations from public databases are incorrect, which are known to be far too common.

## Conclusions

In the current study, we have compared eight different methods for PTIGS-based DNA barcoding analyses. We have also developed tools that would facilitate the retrieval and annotation of PTIGS sequences. All these tools have been integrated into a web server. To our knowledge, this web server represents the very first that supports PTIGS-based DNA barcoding analyses. It would strongly promote the PTIGS-based DNA barcoding applications.

## Availability and requirements

The web server is available at http://psba-trnh-plantidit.dnsalias.org with no requirements for both non-profit and commercial users.

## List of abbreviations

PTIGS: *psb*A-*trn*H Intergenic Spacer; DNFP: Di-Nucleotide Frequency Profile

## Competing interests

The authors declare that they have no competing interests.

## Authors’ contributions

CL designed the study, implemented the Edit distance calculation method and set up the frame work for the web server, wrote the paper except the background section. TG wrote the background section. DL implemented the DNFP method and carried out the comparison of the eight different methods. XHP, JYS, HY, JPH, ZHL, KJ and XJG tested the web server. HL and SLC critically reviewed the article.

## Supplementary Material

Additional file 1 **Sequences used in this study** This file contains all sequences in fasta format used in this study. Click here for file

Additional file 2 **Taxon ids for sequences used in this study** This file contains the mapping between the taxon ids from NCBI and their corresponding sequence accession numbers. Click here for file

Additional file 3 **Exemplar sequences used in method evaluation** This file contains the three sequences used as the example to demonstrate that Blast+P distance method is advantageous to the Blast methods. Click here for file
