# Supplementary material for: PTIGS-IdIt, a system for species identification by DNA sequences of the psbA-trnH intergenic spacer region
Source: BMC Bioinformatics. 2011 Nov 30;12(Suppl 13):S4. doi: 10.1186/1471-2105-12-S13-S4 (PMC3278844; doi:10.1186/1471-2105-12-S13-S4)
Supplement: Additional file 3 — Exemplar sequences used in method evaluation This file contains the three sequences used as the example to demonstrate that Blast+P distance method is advantageous to the Blast methods. [file 1471-2105-12-S13-S4-S3.doc]

Supplementary Figure 1

The three sequences used in the example to demonstrate why the Blast+P methods having better discriminatory power than the Blast method.

>bc_129213_EF590731

AGCTTCTGTTGAAGCTCCTTCTGTAAACGGTTAATATTTTTATTAGCTTTTCTTTTATTATTAAAAAAATTATAATAAAA

ATAACAACTCGGTCAAAATCTTAAAAATGAAAAAAATTTAAGATAAGGATAACAACTTATAAAAAAAGAATGACCTTAGA

GATTTTAAGTCTCTAAGGTCATTCTTTTTATATATAT

>bc_3213_EF590730

AGCTTCTGTTGAAGCTCCTTCTGTAAACGGTTAATATTTTTATTAGCTTTTCTTTTATTATTAAAAAAAATTATAATAAA

AATAATAACTCGGTCAAAATCTTAAAAATGAAAAAAATTTAAGATAAGGATAACAACTTATGAAAAAAGAATGACCTTAG

AGATTTTAAGTCTCTAAGGTCATTTTTTATATATATATATTTTTTTTTGAAAAATGGCGGACGT

>bc_129213_GQ248374

ACAACTTCCCTCTAGATTTAGCTTCTGTTGAAGCTCCTTCTGTAAACGGTTAATATTTTTATTAGCTTTTCTTTTATTAT

TAAAAAAATTATAATAAAAATAACAACTCGGTCAAAATCTTAAAAATGAAAAAAATTTAAGATAAGGATAACAACTTATA

AAAAAAGAATGACCTTAGAGATTTTAAGTCTCTAAGGTCATTCTTTTTATATATATATTTTTTTTATATAAAAATGGCGG

ACGTGGCCAAGTGGATTAAGGCAG

Supplementary Figure 2

The alignment of the three sequences that were found to be the top best hit of the query sequences using Blast. The High Scoring Pair (HSP) regions are underlined. The three sequences are 100% identical in the underlined region.

bc_129213_EF590731 -------------------AGCTTCTGTTGAAGCTCCTTCTGTAAACGGT

bc_129213_GQ248374 ACAACTTCCCTCTAGATTTAGCTTCTGTTGAAGCTCCTTCTGTAAACGGT

bc_3213_EF590730 -------------------AGCTTCTGTTGAAGCTCCTTCTGTAAACGGT

*******************************

bc_129213_EF590731 TAATATTTTTATTAGCTTTTCTTTTATTATTAAAAAAA-TTATAATAAAA

bc_129213_GQ248374 TAATATTTTTATTAGCTTTTCTTTTATTATTAAAAAAA-TTATAATAAAA

bc_3213_EF590730 TAATATTTTTATTAGCTTTTCTTTTATTATTAAAAAAAATTATAATAAAA

************************************** ***********

bc_129213_EF590731 ATAACAACTCGGTCAAAATCTTAAAAATGAAAAAAATTTAAGATAAGGAT

bc_129213_GQ248374 ATAACAACTCGGTCAAAATCTTAAAAATGAAAAAAATTTAAGATAAGGAT

bc_3213_EF590730 ATAATAACTCGGTCAAAATCTTAAAAATGAAAAAAATTTAAGATAAGGAT

**** *********************************************

bc_129213_EF590731 AACAACTTATAAAAAAAGAATGACCTTAGAGATTTTAAGTCTCTAAGGTC

bc_129213_GQ248374 AACAACTTATAAAAAAAGAATGACCTTAGAGATTTTAAGTCTCTAAGGTC

bc_3213_EF590730 AACAACTTATGAAAAAAGAATGACCTTAGAGATTTTAAGTCTCTAAGGTC

********** ***************************************

bc_129213_EF590731 ATTCTTTTTATATATAT---------------------------------

bc_129213_GQ248374 ATTCTTTTTATATATATATTTTTTTTATATAAAAATGGCGGACGTGGCCA

bc_3213_EF590730 ATTTTTTATATATATATATTTTTTTTTGA--AAAATGGCGGACGT-----

*** *** *********

bc_129213_EF590731 ---------------

bc_129213_GQ248374 AGTGGATTAAGGCAG

bc_3213_EF590730 ---------------
